# Supplementary material for: Towards developing a beef meat export oriented policy in Tanzania: -Exploring the factors that influence beef meat exports-
Source: PLoS One. 2022 Jun 17;17(6):e0270146. doi: 10.1371/journal.pone.0270146 (PMC9205477; doi:10.1371/journal.pone.0270146)
Supplement: Attachment — Submitted filename: Response to Reviewers.pdf [file pone.0270146.s002.pdf]

Response to the Reviewers' Comments

We thank the editor and reviewers for taking their time to read and give their comprehensive and constructive comments, which have improved our manuscript. Below; we provide a point-by-point response to your comments and suggestions and how each one has been addressed in the revision.

Response to Reviewer 1 Comments

Comment 1: The manuscript is technically solid, the data obtained by the authors support the investigation and its conclusions, and thus the study exhibits novel research findings. What was particularly applauded was the careful examination of the causes for low exports, especially given that with the minor export constraints, Tanzanian beef meat exports could expand proportionately. According to the given data, the authors have not previously published this study. The paper's format is logical, beginning with an overview of the problem and progressing through the methods and sources of data collecting. The article spans the years 1985 through 2020 and employs descriptive and inferential statistics, as well as regression (econometric) analysis models, to examine the data. The studies are carried out to a high technical degree and are sufficiently detailed within the publication, demonstrating that the analysis was carried out appropriately and systematically. The authors have included all data supporting their findings in their publication in tables, along with explanations for those tables. Additionally, the conclusion is given appropriately, the evidence supports the conclusions, and the policy implications are reasonable and based on the findings. The research complies with all applicable ethical and research integrity standards, and the work adheres to appropriate reporting criteria and community standards for data access. The only (relatively minor) issue with the most recent version of the paper is that it contains a few typos, extra spaces before percentage characters, and missing spaces or letters (e.g. Tanzanian government, p. 10), but my overall recommendation is to publish the paper following these minor revisions to correct the typos.

Response: We thank the reviewer for these comments. We appreciate the compliment from the reviewer that the manuscript is technically solid, the data obtained by the authors support the investigation and its conclusions, and thus the study exhibits novel research findings. However, a few typos, extra spaces before percentage characters and missing spaces or letters have been corrected.

Response to Reviewer 2 Comments

Comment 1: The paper's main goal is to discover the factors which influence the beef export in Tanzania. The topic is somewhat original; however, its statistical approach is, indeed. So I accept it as an original submission.

Response: We thank the reviewer for seeing the useful information contained in our manuscript and accept it as an original submission.

Comment 2: The literature review should be a separate part of the paper. Now it is integrated with the introduction but not enough and isn't made appropriately. So the literature review part should be extended, touching on the general issues of the beef value chain and its export significance.

Response: We thank the reviewer for this comment. We are sorry for the shortcomings in our previous manuscript. The literature review part has been extended, touching on the general issues of the beef value chain and export significance.

Comment 3: The methodology description is too technical, and hard to follow logically - it should be explained, why the selected method was chosen for the research (worth considering starting the section with the conceptual framework).

Response: We thank the reviewer for this important comment. We have followed the suggestions from the reviewer to indicate why the method was chosen for the research in our current manuscript. Again we have considered starting the section with the conceptual framework.

Comment 4: The source for Table 2 is not indicated.

Response: We thank the reviewer for yet another important reminder. We have indicated the Sources in Table 2.

Comment 5: Figure 1 is missing, we just can see the title

Response: We thank the reviewer for this comment. Figure 1 is included in the manuscript.

Comment 6: The abstract should be rewritten to be more compact and comprehensive?

Response: We thank the reviewer for this comment. We have rewritten the abstract to be more compact and comprehensive. We hope it is clear now.

Comment 7: Conclusions are too short and do not contain implications
Response: We thank the reviewer for this comment. We double-checked and expanded the conclusions, as well as added implications.

Comment 8: Limitations of the research should be indicated
Response: We thank the reviewer for this comment. We have indicated the Limitations of the research in our revised manuscript.
